# Classroom observation data collected to document the implementation of physics competence-based curriculum in Rwanda

**DOI:** 10.1016/j.dib.2021.107055

**Published:** 2021-04-20

**Authors:** Kizito Ndihokubwayo, Jean Uwamahoro, Irénée Ndayambaje

**Affiliations:** African Center of Excellence for Innovative Teaching and Learning Mathematics and Science (ACEITLMS), University of Rwanda College of Education (URCE), Kayonza, P.O Box 55 Rwamagana, Rwanda

**Keywords:** RTOP, COPUS, Learning and teaching physics, Classroom observation data, Rwanda

## Abstract

Classroom observation has played a role in documenting classroom practices to improve teaching and learning outcomes. This dataset allows teachers, researchers, and educational policymakers to reanalyze it depending on the interest variables and understand Rwanda's current physics education. The data was collected among qualified teachers from selected schools in Kigali city and rural eastern province in Rwanda. Classroom observation data were collected using the Reformed Teaching Observation Protocol (RTOP) and Classroom Observation Protocol for Undergraduate STEM (COPUS). This data article describes the collected data, research design undertaken, and methods used to collect and analyze them.” The data collected are valid and reliable due to standard instruments used and consistent agreement between classroom observers. A step-by-step procedure on collecting and analyzing COPUS data and checking interobserver reliability has been detailed and informed in supplementary materials.

**Specifications Table**

**Table:** 

Subject	Physical sciences
Specific subject area	Physics education
Type of data	Table Graph Figure
How data were acquired	Classroom observation was done using the Reformed Teaching Observation Protocol accessible at https://www.physport.org/assessments/assessment.cfm?A=RTOP and Classroom Observation Protocol for Undergraduate STEM accessible at https://www.physport.org/assessments/assessment.cfm?I=66&A=COPUS
Data format	Raw Analyzed
Parameters for data collection	Observed teachers are qualified to teach physics and have received training on competence-based curriculum implementation. Before data collection, ethical permission was granted, training on how to use RTOP and COPUS was conducted, and interobserver reliability was checked.
Description of data collection	Upon getting a preferable reliability agreement between observers, three observers interchangeably shared classes to observe. The observer sat in a classroom, observed how the teacher was teaching, how learners were learning. For RTOP, Observer took notes during the lesson delivery. After class, the observer fills the RTOP form by rating each of 25 statements on a 0–4 scale. For COPUS, observers recorded on printed sheets the practices done by students and their teachers in every 2 min interval.
Data source location	Institution: the authors are affiliated with the African Center of Excellence for Innovative Teaching and Learning Mathematics and Science (ACEITLMS) at the University of Rwanda College of Education (URCE) City/Town/Region: Kayonza Country: Rwanda Latitude and longitude (and GPS coordinates, if possible) for collected samples/data: the data were collected from schools located in Eastern Province and Kigali city.
Data accessibility	The RTOP data are available at https://data.mendeley.com/datasets/5hbwt43yk3/1[1] The COPUS data are available at https://data.mendeley.com/datasets/tydm5585bj/2[2] The repository contains three-four files, where three of them are for reliability testing while one is for raw and analyzed data. These are: (1) COPUS Reliability Testing - data collected from Rwanda 2019 - detailed procedural reliability analysis (MS Excel file) (2) COPUS Reliability Testing - data collected from Rwanda 2019 (SPSS file) (3) COPUS Reliability Testing - data collected from Rwanda 2019 - output of Kappa statistics (SPSS file) (4) COPUS Data - Grade10&11 Rwandan physics classroom 2019 – Ndihokubwayo (MS Excel file) [2]
Related research article	K. Ndihokubwayo, J. Uwamahoro, and I. Ndayambaje, “Implementation of the Competence-Based Learning in Rwandan Physics Classrooms: First Assessment Based on the Reformed Teaching Observation Protocol,” *EURASIA J. Math. Sci. Technol. Educ.*, vol. 16, no. 9, pp. 1–8, 2020, doi: 10.29333/ejmste/8395. K. Ndihokubwayo, J. Uwamahoro, and I. Ndayambaje, “Usability of Electronic Instructional Tools in the Physics Classroom,” *EURASIA J. Math. Sci. Technol. Educ.*, vol. 16, no. 11, pp. 1–10, 2020, doi: 10.29333/ejmste/8549.

## Value of the Data

• The dataset is available for teachers. Teachers will self-evaluate by comparing their activities to those of their students. They will evaluate where the improvement is needed and track change accordingly.
• The dataset is available for policymakers or educational evaluators. These data will help them to evaluate their programs implemented, such as the competence-based curriculum and its active learning techniques expected to be used by teachers.
• The dataset is available for research practitioners. This data article will help them effectively use the RTOP and COPUS tool through a detailed procedure on conducting training, checking reliability, collecting data, and analyzing data. They can follow the data outcome and further analyze them among other variables such as school location and type of school, and grade level or physics content.

## 1 Data Description

### 1.1 RTOP data

The data was collected by observing 42 classes from nine teachers teaching in six schools. The data collected were analyzed using MS Excel (see http://dx.doi.org/10.17632/5hbwt43yk3.1). The raw data are recorded in the “RTOP Raw Data” sheet, while analysis is made in the “RTOP Analysis-1” and “Analysis-2” sheets. The data are ordinal data types as they are ranked on a 5-point scale, from 0 to 4 scores. The RTOP statements are arranged vertically, while classes are arranged horizontally. Data are recorded in numbers, and 0, 1, 2, 3, or 4 is recorded under each class corresponding to a specific RTOP statement (see “RTOP Raw data” sheet). The analysis from top to bottom shows each statement's total scores across all 25 RTOP statements, while the analysis from left to right shows the total scores of each statement across all 42 classes (see “RTOP Analysis-1” sheets). Thus, the maximum total score across all 25 RTOP statements is 100 (when the maximum 4-scores are scored to each of 25 RTOP statements) for individual class while the maximum total scores across all 42 classes are 168 (when the maximum 4-scores are scored in each of 42 classes) for individual RTOP statement.

The data are recorded under four variables (Table 1). These are (a) location of school (two urban and four rural schools), (b) type of school (two day and four boarding schools), (c) grade level (21 classes were recorded from senior four or grade-10, while other 21 classes were observed from senior five or grade-11), and (d) teaching topics (25 observed classes, teachers were teaching optics related topics while in other 17 observed classes, teachers were teaching other lessons, not related to optics). Thus, 27 classes were observed from schools located in Kigali city, while 15 were observed in the eastern province. Likewise, 35 classes were observed from schools where students stay at school (day and night) while seven classes were observed in schools where students go to school in the daytime and spend the night at their home [4].

**Table 1:** Classification of observed lessons with RTOP.

			Grade level		
Location of school	Type of school	School code	Senior-4	Senior-5	Total
Urban	Boarding	SCH-1	13	12	25
	Boarding	SCH-3	2	0	2
Rural	Boarding	SCH-5	2	2	4
	Day	SCH-6	1	1	2
	Boarding	SCH-7	1	3	4
	Day	SCH-8	2	3	5
Total class			21	21	42
Optics lessons					25
Other lessons					17

The analysis of these variables is computed using descriptive statistics—means and standard deviations—and displayed in the “RTOP Analysis-1” sheet. This sheet also shows the analysis of RTOP components (sub-themes) [3]. The descriptive analysis of each RTOP statement and the factor analysis of six groups is computed from the “RTOP Analysis-2” sheet.

### 1.2 COPUS data

We observed a total of 62 lessons from 11 teachers from Rwanda in 2019 using COPUS. These teachers are qualified to teach physics in secondary schools. These teachers are from eight schools purposively sampled to include rural and urban schools and day and boarding schools that accommodate physics subjects. Data we collected by three observers, and all observers did not observe the same lessons due to the time limit. We observed optics-related lessons, and these lessons were to be covered in only three months (January–March 2019). The COPUS developers advise researchers to observe at least three lessons from each teacher teaching one subject to the same students in order to track well and understand teacher' practices. In fact, you cannot evaluate someone in a single observation depending on several characteristics, such as the nature of the content, environment, and psychological condition. Table 2 shows that 53 lessons achieved this objective. Thus, teacher four (T4), T5, T6 Grade 10 lessons, and T7 and T11 Grade 11 lessons would not be considered for analysis.

**Table 2:** **Classification of observed lessons with COPUS.** The number in parenthesis indicates the number of students in the classroom, “-” indicates that the teacher does not teach in that grade, while “0” shows that teacher teaches in that grade, but no lesson was observed there.

Teacher's code	Teacher's gender	School code	Type of school	Location of school	Observed lessons in Grade 10	Observed lessons in Grade 11
T1	Female	Sch1	Boarding	Urban	7(29)	11(43)
T2	Male	Sch1	Boarding	Urban	6(44)	–
T3	Male	Sch1	Boarding	Urban	–	4(30)
T4	Male	Sch2	Day	Urban	1(36)	0
T5	Male	Sch3	Boarding	Urban	2(19)	0
T6	Female	Sch4	Day	Urban	2(12)	3(15)
T7	Male	Sch5	Boarding	Rural	4(19)	2(29)
T8	Male	Sch6	Day	Rural	4(18)	–
T9	Male	Sch6	Day	Rural	–	3(44)
T10	Male	Sch7	Boarding	Rural	5(19)	3(19)
T11	Male	Sch8	Day	Rural	3(12)	2(12)
Total lessons					34	28

## 2 Experimental Design, Materials and Methods

### 2.1 Reformed teaching observation protocol

The data shared in this article was collected from January to March 2019 using the Reformed Teaching Observation Protocol (RTOP) developed by Sawada et al. [7,8]. RTOP is an excellent tool to document any science or mathematics lesson's practices. It comprises background information and the contextual background and activities [8]. In the background information section, the observer fills information about the teacher's characteristics, such as name, experience, and grade level. In the contextual background and activities, the observer rate-the occurrence of-each of 25 RTOP statements from 0 to 4. It means that if a particular activity did never happen, the observer scores it 0. When the activity happened entirely and throughout the whole class, this is scored the maximum score of 4. Before observing classes, the chance-corrected inter-observer reliability was checked [3]. The researchers informed teachers and students whenever observation would be taken place. Observer sat in classroom and observed class without interaction, took notes, and fill out the printed protocol sheet (see https://www.physport.org/assessments/assessment.cfm?A=RTOP) using pen at the end of class by evaluating what was done during teaching and learning time.

### 2.2 Classroom observation protocol for undergraduate stem

The Classroom Observation Protocol for Undergraduate STEM (COPUS) was developed by Smith et al. [5]. Through the Carl Wieman Science Education Initiative at the University of British Columbia in 2013 and designed to collect data from STEM classrooms specifically. COPUS is a valid and reliable tool for collecting data related to what is happening in the classroom. Though it was designed to document what is happening at the undergraduate level, later it was found that it may also be used in graduate and secondary school levels [6]. It has 28 codes; among them, 13 describe students doing, 12 describe teacher/instructor doing, while three codes describe how the teacher engages his/her students. However, the codes related to student engagements are not mandatory. A detailed description of each code may be found here https://trestlenetwork.ku.edu/wp-content/uploads/2019/01/COPUS_Code_Descriptions_UpdatedJanuary2019.pdf.

The data are analyzed by MS Excel using the COPUS visualization template found at https://tep.uoregon.edu/files/copus_with_visualization.xlsx. This template has three main sheets; (a) COPUS data entry, (b) percent activities graphs, and (c) percent of time intervals graph. From the field, you fill data in the COPUS data entry sheet. The sheet contains automatic formulas that analyze the data entered directly by counting the number of times a code was observed and by counting the number of 2-min segments any code appeared. The provided template can be entered in a lesson of up to 110 min. Our records were more than this. So, we extended the formulas to accommodate all of our data. Below the COPUS data entry sheet table (see our file titled “COPUS Data-Grade10&11 Rwandan physics classroom 2019-Ndihokubwayo” [1]), each code is summed up to provide the number of times that code was checked; see Row 2190. The total scores across all students codes are located on B2191, and one across all teacher/instructor codes is located on O2191, while one across engagement level codes is located on AA2191. The right side of the table of data (column AE) shows where any code was checked across a 2-min interval. When one or many codes was/were checked, “1” score is marked, while a 2-min interval that none of the code was checked, then a “0” score is marked. Thus, the sum in this column is located on AE2191 and shows the number of time intervals.

The COPUS data are analyzed in two ways. One way is the percentage of activities (relative abundance), while another way is the percentage of time intervals (relative frequency). Analyzing the percentage of activities is vertical. Our data shows that 2478 are total codes checked under students' codes while 3325 are total checks under teacher's codes. The percent of activities graphs displays these data and shows the computed percentage of each code. For instance, the percentage of occurrence of listening “L” code is 918 over the total of all students code counts; thus, “L” happened 918/2478 or 37% of all students activities. Note that 13 students' activities (codes) share 100%; thus, listening shares 37% of all activities. The graph of all activities is generated by considering each code's percentage. However, [2] suggested combining codes such Lec, RtW, and D/V as “Presenting,” FUp, PQ, CQ, AnQ, MG, and 1o1 as “Guiding,” Adm as “Admin,” and W and *O* as Other” for teacher's activities while L as “Receiving,” AnQ, SQ, WC, and SP as “Talking to Class,” Ind, CG, WG, and OG as “Working,” and *O* and *W* as Other were combined for students activities. The grouping of these codes easy to compare instructional styles and maybe formulated depending on the research focus, such as the investigation of clicker questions [4], active learning techniques [3], instructional style [5], and so on.

Analysis of percentage of the time interval is horizontal. Our data were recorded into 1940 2-minute segments. The percent of time intervals graph sheet analyses the occurrence of each code percentage of 2-min intervals in which each code occurred. Thus, for 918 occurrences of listening “L” code will be counted on 1940 segments instead of 2478 total of all students codes. Thus, “L” occurred 918/194 or 47% of all 2-min intervals. This analysis is better than the one of percent of activities because it considers both activities done across other activities and considers time spent performing it. Thus, listening has taken 47% of 1940 2-minute intervals, among other activities. Marilyne Stains and colleagues have developed a COPUS profile analyzer (http://www.copusprofiles.org), an automatic analyzing COPUS data. For a detailed how-to compute reliability among two or more observers, please attached supplementary materials.

## Ethics Statement

Before collecting data, the research proposal was sent to the research and innovation unit at the University of Rwanda College of Education (URCE) for ethical evaluation. After reviewing the research proposal, ethical clearance was granted [Ref: 01/P-CE/483/EN/gi/2018] and used to ask permission at district levels. Permissions were requested in two districts in Kigali city and two districts in the eastern province to access their schools. School leaders facilitated us and introduced us to our target research participants-teachers and students-who were requested to sign the consent forms after explaining the research purpose and assuring them voluntary participation. Informed consent of all these participants has been obtained.

## CRediT Author Statement

**Kizito Ndihokubwayo:** Conceptualization, Methodology, Data curation, Formal analysis, Writing original draft; **Jean Uwamahoro:** Conceptualization, Visualization, Writing review & editing, Supervision; **Irénée Ndayambaje:** Conceptualization, Validation, Writing review & editing, Supervision.

## Declaration of Competing Interest

The authors declare that they have no known competing financial interests or personal relationships which have or could be perceived to have influenced the work reported in this article.

## Appendix Supplementary materials

Image, application 1

Image, application 2
