# Supplementary figures and images for: Classroom observation data collected to document the implementation of physics competence-based curriculum in Rwanda
Source: Data Brief. 2021 Apr 20;36:107055. doi: 10.1016/j.dib.2021.107055 (PMC8113727; doi:10.1016/j.dib.2021.107055)

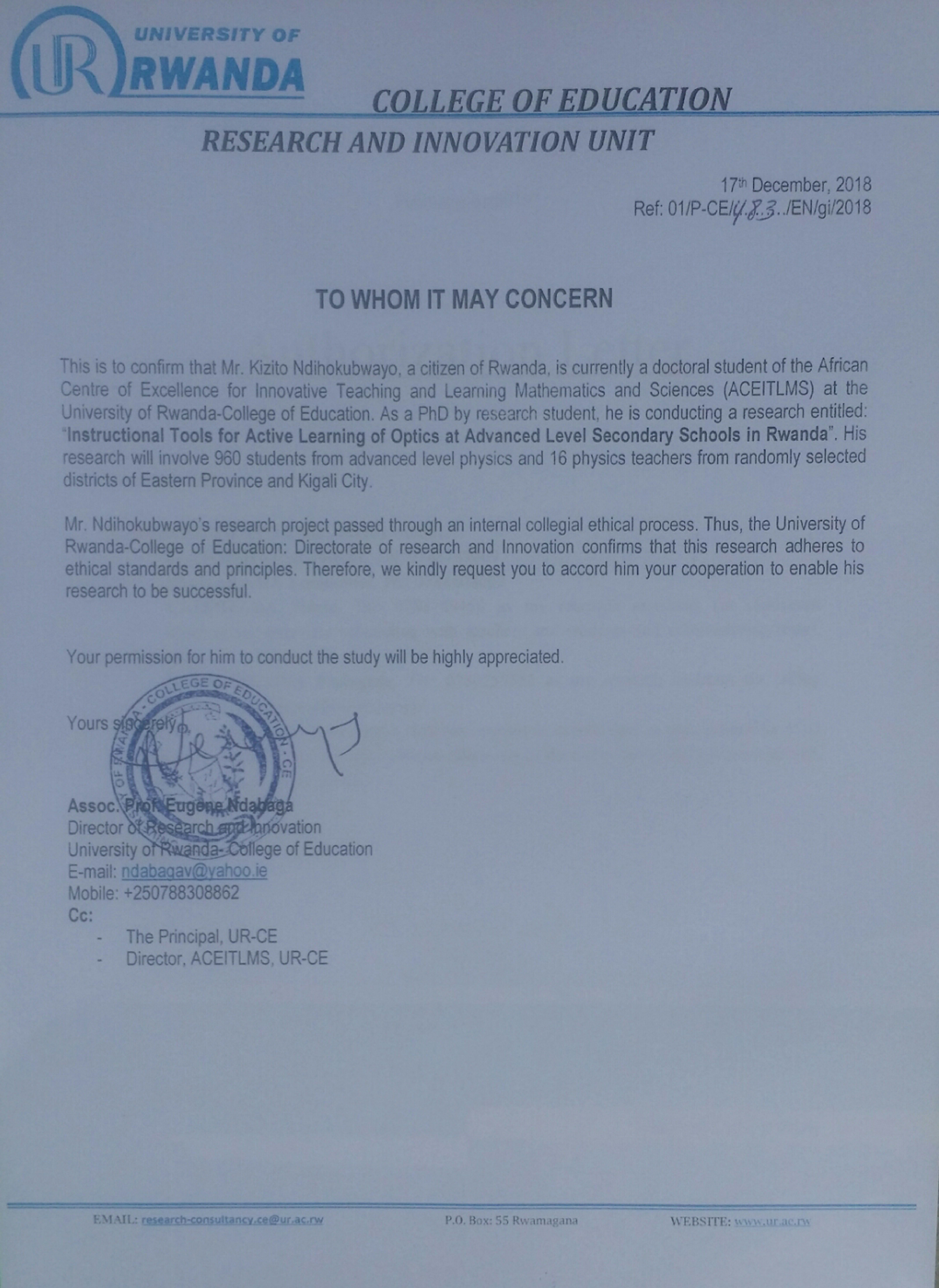

Supplement: Supplementary file 1 [file mmc1.jpg]
