# Supplementary material for: Classroom observation data collected to document the implementation of physics competence-based curriculum in Rwanda
Source: Data Brief. 2021 Apr 20;36:107055. doi: 10.1016/j.dib.2021.107055 (PMC8113727; doi:10.1016/j.dib.2021.107055)
Supplement: Supplementary file 2 [file mmc2.docx]

**How to Collect and Analyze Classroom Observation Protocol for Undergraduate STEM (COPUS) Data**

Classroom observation provides tremendous data on what behaviors teachers and students are displaying in the classroom. This text presents a detailed procedure for collecting and analyzing Classroom Observation Protocol for Undergraduate STEM (COPUS) Data. We show how training is done before entering the classroom, a step-by-step procedure on how to check similarity coding between observers has been detailed and informed. This dataset is essential for practitioners and researchers who undertake the COPUS tool for theoretical implication. It is also important to teachers and policymakers for practical implications.

The data are uploaded in the Mendeley Repository at this link: <https://data.mendeley.com/datasets/tydm5585bj/2> [1]

Instructions for accessing these data: the data are open and available to anyone who wants to learn how to check COPUS reliability and anyone who wants to use the data.

The repository contains three-four files, where three of them are for reliability testing while one is for raw and analyzed data. These are:

1. COPUS Reliability Testing - data collected from Rwanda 2019 - detailed procedural reliability analysis (MS Excel file)
2. COPUS Reliability Testing - data collected from Rwanda 2019 (SPSS file)
3. COPUS Reliability Testing - data collected from Rwanda 2019 - output of Kappa statistics (SPSS file)
4. COPUS Data - Grade10&11 Rwandan physics classroom 2019 – Ndihokubwayo (MS Excel file)[1]

**COPUS Training**

A 2-hour training should be done before using COPUS [2]. A detailed description of each COPUS code may be found here <https://trestlenetwork.ku.edu/wp-content/uploads/2019/01/COPUS_Code_Descriptions_UpdatedJanuary2019.pdf>

On 7^th^ January 2019, the first author and one research assistant attended online training delivered by the Transforming Education, Stimulating Teaching and Learning Excellence (TRESTLE) group at the University of Kansas (KU). This was a two-hour training where the trainers first introduced the meaning of COPUS codes, and then after, they played a video so that participants observe and code activities that are happening in each 2-min. After every 2 minutes, the video was stopped, and participants share plenary what COPUS codes they have coded. After online training, the first author trained another research assistant on how to collect data using COPUS in the same way trainers from KU did, but this was not online. During his student exchange program, the first author has attended physical training on how to analyze the COPUS data at KU between 6^th^ and 27^th^ September 2019.

**Reliability testing**

Before visiting schools for data collection using the COPUS, we needed first to check the observers' agreement. Checking agreement between observers gives confidence that data collected are reliable and valid due to the constantly observed classroom practices. Thus, observers should observe practices in quite the same way, and the rated code should be accurate across all observers. We have observed a recorded video of a physics class and then calculated the agreement between observers. During the observation, observers filled or checked a printed COPUS form (see <https://cwsei.ubc.ca/sites/default/files/cwsei/resources/tools/COPUS_protocol.pdf>) by pen by putting a check or a tick under any code (a practice happening in the classroom) during each of 2-min intervals. After watching the video, we recorded the data into the same form (MS Excel version) by putting "1" where the observer ticked or put a check. Where the observer did not tick, remains empty-filled or filled with "0" for easy analysis.

The MS Excel and SPSS files present the data of reliability. The link is shown in the “Data accessibility” under the Specification Table. MS Excel file is titled "COPUS Reliability Testing - data collected from Rwanda 2019 - detailed procedural reliability analysis," while SPSS files are titled "COPUS Reliability Testing - data collected from Rwanda 2019" and "COPUS Reliability Testing - data collected from Rwanda 2019 - output of Kappa statistics." Please access these files here <https://data.mendeley.com/datasets/tydm5585bj/2> or [1].

The first SPSS file data are similar to the data in the MS Excel file (from Raw 3 to Raw 24). These are data from two observers observing one lesson of 40 minutes—20 segments of 2-min—across all 28 COPUS codes. The data are arranged side by side, where the first column for each code is the coded data from observer-1 while the next column is for observer-2. For instance, column D – Row 4 is L – observer-1, while column D – Row 4 is L – observer-2 in MS Excel file [1].

Following the analysis steps in the MS Excel file “reliability detail procedure” [1], Row 28 – 51 shows the analysis of agreement between two observers. If you click on any cell under any code, you will find how we merged the code checked by observer-1 or observer-2. We summed the observed codes from both observers. Thus, when observer-1 and observer-2 both checked the box (or observed that a certain activity happened), the sum adds up to "2," when only one observer observed that code, the sum adds up to "1," and when none observed a certain activity (or none ticked on a certain code), the sum adds up to "0." Then, we counted how many cells observers put a check (number of times that both observers agreed on each code), how many cells that either observer-1 or observer-2 put a check (number of disagreement between observers), and how many cells none of the observers put a check (agreement on non-appearing of a certain code). Thus, the agreement was the ratio of all agreement (either observed or not observed by all observers) and all possible cells to be put a check—In this case, 20 segments across all 28 COPUS codes—(see Row 55 – 61).

$Agreement (A)=\frac{Cells checked+Cells not checked}{All possible cells}$ $A=\frac{82+447}{560}$ $A=0.94=94\%$

Row 63 – 72 shows a detailed analysis of each code. We symbolized "nc" as all cells where both observers agree (put a check or not, COUNT 2 and 0), "nab" as the number of cells checked either by observer-1 or observer-2 (COUNT 1), and "OA" as observed agreement on each code. For instance, both observers agreed 17 times and disagreed 3 in 20-time segments on "L" code; thus $OA=\frac{17}{20}=0.85=85\% on listening "\text{L" }code$. After computing all 28 codes, their average will be the same as "A" above (see Row 72 of MS Excel file).

Note that an agreement between observers contains some agreement by chance because it shows only the similarities scores; therefore, there is a need to compute Kappa statistics [2] to remove this. See Row 75 – 134 in our MS Excel file shows Cohen's Kappa's analysis between two observers. We deployed data from SPSS (Cohen's Kappa, Row 80, and crosstabulations, Row 91 – 97, Row 107 – 113, and Row 123 – 129) to an MS Excel file to show how to compute the Kappa statistics using MS Excel 2016. The Cohen's Kappa IOR does not count the codes where all the observers agreed without variability. This is when either both observers observed or not observed a code throughout. For instance, both observers did not observe a "CG" code throughout 20 segments. Thus, the agreement is perfect (100%), but no Cohen's Kappa IOR (it cannot be computed). This does not mean that we do not consider Cohen's Kappa IRR wherever we get a perfect agreement. For instance, on "WG" code, there was 100% of agreement between two observers, and the Cohen's Kappa IOR was computed to be "1" or "100%" because although observers got similar checks, this code has variability where some agreements are from observing this codes (checked cells) and from not observing this code (unchecked cells). See column P – Q through Row 5 – 24 for unavailable Cohen's Kappa IOR since the only one-way agreement was found (unobserved code by all observers) on "CG" code. See column R – S through Row 5 – 24 for available Cohen's Kappa IRR due to the fact that two ways agreement was found (some are observed "1" others are unobserved "0" similarly) on "WG" code.

Cohen's Kappa interobserver reliability or Cohen's Kappa IOR is calculated using two major agreements: the observed agreement (OA) and agreement by chance (AC). We have seen how to calculate observed agreement (OA). Agreement by chance (AC) was generated using SPSS data. With the data in the SPSS file (COPUS Reliability Testing - data collected from Rwanda 2019), we checked Cohen's Kappa IRR via "analyze – descriptive statistics – crosstab." Under Crosstabs options "Statistics," check "Kappa" and hit "continue" and "Ok." You will get results similar to those in the SPSS output file (COPUS Reliability Testing - data collected from Rwanda 2019 - output of Kappa statistics). The third table displays measurement agreement (Kappa) values, while the second table displays a crosstabulation between observer-1 and observer-2 coding. We copied the second table and put it in an MS Excel file in order to get data for computing agreement by chance (AC). The crosstabulation shows clearly the number of cells checked or not checked by each of the observers. It even identifies "nab" (it shows the number of cells check by observer-1 that observer-2 did not check and the number of cells checks by observer-2 that observer-1 did not check). An example of calculating AC is presented in Table 1.

| **Listening - observer 1 * Listening - observer 2 Crosstabulation** | | | | |  |
| --- | --- | --- | --- | --- | --- |
| Count | | | | |  |
|  | | Listening - observer 2 | | Total | Proportion |
|  |  | 0 | 1 |  |  |
| Listening - observer 1 | 0 | 13 | 3 | 16 | 0.8 |
|  | 1 | 0 | 4 | 4 | 0.2 |
| Total | | 13 | 7 | 20 |  |
| Proportion | | 0.65 | 0.35 |  |  |

**Table 1. Example of calculating the agreement by chance (AC) between two observers**

Table 1 shows that both observers observed “L” happening four times, did not observe it happening 13 times, and only observer-2 observed it three times while observer-1 did not. Thus, observer-1 observed a total of four times “L” happening and 16 times “L” unhappening. Likewise, observation of “L” happening is totaled to seven; nothing observed is totaled to 13 for the observer-2.

The observed agreement was calculated and found to be 0.85 (85%), and it is located on cell D70 (column D – Row 70) in an MS Excel file. The agreement by chance (AC) was then calculated and found to be 0.59, located on cell D100 in the same file. Using data from table 1 generated by SPSS, we computed the proportions divide by side to get to the AC. Table one shows that both observers checked four cells, unchecked 13 cells. Among 20 total cells, 3 are disagreements between observers. Thus, observer-2 checked in three cells that observer-1 did not check. The proportions were found by taking each of the totals and divide by the total cells (20-time segments). For example, following on the data in table 1; $\frac{13}{20}=0.65$ and $\frac{16}{20}=0.8$

AC is the sum of the product of first proportions and the product of second proportions. For example, following on the data in table 1; $AC=0.65*0.8)+\left( 0.35*0.2 \right)=0.59$

Then, Cohen's Kappa interobserver reliability can be computed by:

$$Cohen^{'}s Kappa IOR (K)=\frac{(Observed Agreement \left( \mathrm{OA} \right)-Agreement by Chance (AC)}{(Expected Agreement \left( 1 \right)-Agreement by Chance (AC)}$$

For example, following the listening "L" code, while the observation agreement between two observers was 0.85, the Cohen's Kappa IOR was $K=\frac{(OA-AC)}{(1-AC)}=\frac{(0.85-0.59)}{(1-0.59)}=0.63.$ Note that the possible agreement is one or 100% as the maximum agreement (expected agreement). From MS Excel Row 138, we presented two other ways of calculating observation agreement manually.
